# The End of the Year

**Published:** 1885-01

**Authors:** 


### EDITORIAL.

### The End of the Year.

Eighteen hundred and eighty-four is just drawing to a close, and the natural and reasonable inclination of every thoughtful person is to retrospection.

The business man has learned that if he will attain success he must take an account of stock, post his books, examine his debits and credits. All this is not only interesting, but it is necessary, that he may know where he stands, and how to shape his course in the future. The merchant is not the only one who will profit by a rigid review of the past. The professional man may derive much good from such a survey. The dentist, as well as any other, may be benefitted by looking over his course of practice for the past year. There are many points upon which it would be well to reflect.

Let each look over his practice during this period with special reference to the results attained, and reckon, if he can, how much of suffering he has been instrumental in relieving, how successfully has disease been combated and health restored. In restoration and prevention how much has been accomplished? Has every case treated received the best that could be done? Has every filling been well and thoroughly made? Is there a consciousness in every instance that better could not have been done? Has every instrument, appliance, preparation, and mode, especially those of recent device, been properly tested and brought into use so far as they may be efficient? Have we made fully available all the resources and opportunities that have been within our reach? Have we co-operated with the profession as fully as we might, for the support of its best interests?

These are a few of the points that every dentist may profitably ponder before entering upon the duties of another year.

Such a review, conscientiously made, will be beneficial to the dentist, and of incalculable value to his patients.

In order to make such a review most useful, it should be carefully written out; and the last pages of an appointed book is the most appropriate place for such a record. Then from year to year comparison could be made which would determine not only the progress of the individual, but also, in a large measure, that of the profession as a whole.

Those who make such a record will find a frequent reference to it not only a source of pleasure but of benefit as well.

